# The use of edible insects in human food

**DOI:** 10.1002/jsfa.70475

**Published:** 2026-01-23

**Authors:** Pamela Barroso de Oliveira, Hugo Calixto Fonseca, Cláudia Regina Vieira, Gleidson Giordano Pinto de Carvalho, Jane Sélia dos Reis dos Coimbra, Bruna Mara Aparecida de Carvalho Mesquita

**Affiliations:** ^1^ Institute of Agricultural Sciences, Federal University of Minas Gerais Montes Claros Brazil; ^2^ Department of Animal Science Federal University of Bahia Salvador Brazil; ^3^ Department of Food Technology Federal University de Viçosa Viçosa Brazil

**Keywords:** entomophagy, entomotherapy, nutrition, protein, sustainability

## Abstract

The world population is expected to reach approximately 10 billion people by 2050, which will significantly increase global food demand and may lead to agricultural shortages and a higher risk of food insecurity. In this context, this review discusses the potential of insects as alternative sources of animal protein, addressing their nutritional, environmental, and social aspects. More than 2000 insect species have been identified as safe for human consumption, offering a wide range of nutrients, including proteins, lipids, minerals, and vitamins at different life stages such as eggs, larvae, pupae, and adults. The fat content of edible insects ranges from 2% to 62%, with a predominance of unsaturated fatty acids, which can account for up to 75% of total fatty acids. Protein content varies between 20 and 76 g per 100 g of dry weight. In addition to their nutritional value, insect‐based food production presents several environmental advantages, including lower water consumption, reduced greenhouse gas emissions, and higher feed conversion efficiency. Beyond human consumption, insects have also been traditionally used for medicinal and therapeutic purposes. Continued research and increasing consumer acceptance may enable insects to play a crucial role in transforming the food industry and contributing to a more sustainable and resilient global food system. © 2026 The Author(s). *Journal of the Science of Food and Agriculture* published by John Wiley & Sons Ltd on behalf of Society of Chemical Industry.

## INTRODUCTION

Population growth is a global concern, especially in relation to the increased demand for food. Projections indicate that the world population could reach approximately ten billion people in less than 30 years. This growth will have a significant effect on food resources, especially on the demand for animal proteins, whose demand could double. This can result in a shortage of agricultural resources and increase the risk of hunger; therefore, adopting sustainable approaches to food production is critical to ensure food security for all.^1^

Owing to their lower environmental impact than that of animal proteins, which include lower greenhouse gas emissions and lower water consumption, many consumers opt for vegetable products. Concern about the welfare of animals and the positive effects on health also motivate many consumers to choose plant‐based products. According to Huang *et al*.,^2^ the search for non‐animal protein alternatives has focused mainly on legumes, such as peas and soybeans, because they have a more complete nutritional profile than other plant sources do. However, there are several negative points, such as a deficiency of essential amino acids, which makes it important to combine different plant sources, such as grains, cereals and legumes, to ensure adequate intake of these amino acids and lower digestibility because they contain more fiber and antinutritional compounds.^3^

A proposed solution to address food shortages resulting from population growth is the use of insects as a source of nutrients to ensure an adequate supply of macronutrients and micronutrients. These organisms stand out as excellent sources of protein, amino acids, lipids, minerals and vitamins. This alternative seeks to overcome the limitations of plant proteins, offering a more nutritious option with less impact on the environment. The objective is to partially replace conventional animal proteins with these sustainable sources, as insects are highly nutritious and abundant. It is estimated that there are approximately 2000 edible species out of more than one million known species, considering their different stages of development.^4^

Edible insects have the potential to establish themselves as fundamental foods in the future on a global scale. In addition to being nutritional, they have less impact on the environment, help preserve natural resources, reduce climate change and require fewer resources, such as water and land, than those necessary for cattle and poultry. They can be raised in small spaces and reproduce quickly, which makes them a viable option for large‐scale production.^5^, ^6^ Among the known insect species, those indicated as the most promising for production on an industrial scale are common house fly larvae, silkworms, yellow mealworms and black soldier flies, as cited by the Food and Agriculture Organization of the United Nations (FAO).^7^

According to Binconcini‐Júnior *et al*.,^8^ the market for edible insects is expanding continuously in Brazil. They can be consumed raw or processed in various ways, such as roasting, frying, cooking or extrusion. Insects are included in the diet mainly as components of several products. However, the lack of specific regulations represents a barrier to its wider adoption in the food industry as noted by the Brazilian Health Regulatory Agency (ANVISA), of 9 March 2022.^9^ Azzollini *et al*. noted that, in response to an email sent to ANVISA about the regularization procedures, companies interested in using insects in food production can request a safety assessment from the agency for these products.^10^, ^11^

The objective of this study is to review the consumption of insects as a potential replacement for animal protein, highlighting nutritional, environmental and social aspects.

## METHODOLOGY

This manuscript is structured as a narrative review. The objective was to provide an integrative and critical discussion of the current literature on edible insects for human food, encompassing nutritional value, processing, food safety, regulatory frameworks and potential therapeutic applications. Rather than applying a systematic review protocol, the present work synthesizes evidence from peer‐reviewed articles, regulatory documents and authoritative reports to identify consistencies, limitations and knowledge gaps in this rapidly evolving field.

The literature search was performed in the Scopus, Web of Science, and Google Scholar databases. The search covered articles published between January 2013 and April 2024 in English, focusing on studies addressing nutritional, environmental, safety, technological, and sociocultural aspects of entomophagy. The searches used specific combinations of keywords, including entomophagy, sustainability, alternative sources, protein, nutrition and entomotherapy. The inclusion criteria were as follows: studies on edible insect species intended for human consumption; and evaluation of nutritional composition, processing methods, safety, environmental benefits, or consumer acceptance. The exclusion criteria were as follows: animal feed studies, non‐English publications, conference abstracts, or inaccessible full texts.

## THEORETICAL FRAMEWORK

### Entomophagy

The practice of ingesting insects for human consumption, called entomophagy, has played an important role in the human diet throughout history; this process is used to obtain protein and caloric nutrients but is also influenced by cultural factors. Jongema estimated that at least two billion people worldwide practice entomophagy regularly, and in approximately 110 countries, insects are consumed as an essential component of the diet, with more than 2000 species identified as safe for human consumption.^12^

Although it is considered an exotic food practice and is not widely accepted in the West, entomophagy has gained prominence in recent years, especially in research. This growing interest is due to the potential of insects as alternative raw materials to conventional sources in food production. Entomophagy may also be a viable solution for global food security, as it offers the necessary amount of food to meet physiological needs, in addition to the quality and nutritional value essential for maintaining health.^10^, ^13^

Edible insects play crucial roles as food supplements and may benefit the health of vulnerable populations that are experiencing malnutrition, especially children and breastfeeding mothers, as noted by De and Chattopadhyay, especially in low‐ and middle‐income countries. Child growth and development depend on adequate nutrition in early childhood, which can be maintained only with the correct and balanced intake of food.^14^

Given this scenario, it is essential to adopt sustainable strategies to address food shortages. One of these solutions is the use of insects as ingredients for food fortification to increase their nutritional value or as food supplements to meet the demands of micronutrients and macronutrients.^15^

The most popular edible insect species belong to the order Coleoptera, such as beetles, followed by the orders Lepidoptera, which include butterflies and moths, and Hymenoptera, which includes bees, wasps and ants. However, globally, domestic crickets (order Orthoptera) and mealybug beetle larvae (order Coleoptera) stand out as the insects most cultivated exclusively for human consumption.^16^ Consumption can occur at different stages of an insect's life cycle (e.g., larvae, pupae or adults), which is completed in approximately 60 days, and directly affects the nutritional composition, as well as the feeding, sex and origin of the insect.^17^

### Examples of insects used in food

*Bombyx mori*, known as the silkworm, is an insect of great economic importance, with a life cycle that includes eggs, larvae, pupae and adults. Larvae and pupae are valued for their silk production and for their nutritional content, including protein, fatty acid, vitamin and mineral contents. When considered edible, the silkworm is widely consumed in Asian countries such as India, Japan, Thailand and China.^18^ In addition to their high nutritional value, silkworms have health benefits, such as a low sodium‐to‐potassium ratio, which may help reduce the risk of cardiovascular disease, and are rich in selenium, which offers protection against cancer and oxidative stress.^19^, ^20^ Minerals such as calcium, iron and potassium play essential roles in bone health, oxygen transport and blood pressure control. With approximately 30% lipids in dry weight and 55% to 60% protein, the silkworm is a promising source of nutrients and has therapeutic benefits.^21^, ^22^

According to Gkinali *et al*., *Tenebrio molitor*, one of the most promising edible insects, goes through four life stages: eggs, larvae, pupae and adults. The *Tenebrio molitor* eggs hatch between 3 and 9 days after deposition. The larval stage is the longest, lasting from 57 days under controlled conditions to up to 2 years in environments with low temperatures. The larvae then enter the pupal stage, which is the shortest of all developmental stages, lasting between 7 and 48 days, depending on temperature. During this period, the organisms do not feed and gradually develop a rigid exoskeleton. Finally, adults emerge, with this phase lasting between 2 and 3 months.^23^ The larval stage of *Tenebrio molitor*, known as yellow mealworm, is highly valued for its high protein content (37% to 68% of dry weight), which is comparable to that of conventional sources such as meat and soybean, in addition to fat (15% to 50%) and fiber (9% to 19%).^23^ The larval stage is rich in essential minerals such as potassium, calcium, iron and magnesium. Its efficient digestion of organic waste and low amount of chitin increase the bioavailability and digestibility of proteins, which include all amino acids essential for human health. Unsaturated fatty acids such as oleic and linoleic acids are also abundant.^24^, ^25^ However, the adult stage of *Tenebrio molitor* (beetle) has a high chitin content, which limits its nutritional efficacy because of the need for chitinase enzymes for digestion, which are enzymes that are not abundant in the human digestive system.^25^, ^26^ Currently, *Tenebrio molitor* is used as human food and animal feed in several regions of the world, including Africa, Asia, the Americas and Australia.^27^

The black soldier fly (*Hermetia illucens*), a detritivorous insect native to tropical and subtropical regions of the Americas, has attracted increasing interest because of its ability to digest a variety of organic substrates, promoting nutrient recovery and contributing to the reduction in greenhouse gas emissions.^28^, ^29^ The larvae of this insect have an average of 42% highly digestible crude protein and 29% fat in the dry matter. Owing to their high‐quality amino acid profile, larvae are an excellent source of protein and a sustainable alternative for food security.^30^ Compared with fishmeal and soy, these products are particularly rich in valine, histidine and isoleucine, in addition to having higher levels of leucine and lysine.^31^

The domestic cricket (*Acheta domesticus*) is one of the best‐known insect species for human consumption and is widely cultivated in both Eastern and Western countries. Its popularity is due to its ease of cultivation, high growth rate and excellent nutritional profile.^32^ The protein content of crickets ranges from 13% to 77% of the dry matter, with lipids between 20.4% and 29.3% and carbohydrates between 5.50% and 8.32%. It is also rich in essential macrominerals such as sodium, potassium, phosphorus, calcium and magnesium, making it a nutritious alternative to poultry, pork and cattle.^32^, ^33^ In addition to their nutritional value, crickets have beneficial properties, such as anti‐inflammatory, antioxidant, anticancer, antidiabetic, and antiobesity effects, in addition to assisting in muscle building, stimulating the growth of the intestinal microbiota and reducing tumor necrosis factor‐alpha (TNF‐α) levels. Substances such as chitin and chitosan, present in crickets, also help to suppress pathogenic microorganisms in the intestine.^34^

Domestic crickets are the insects with the highest protein content, whereas mealworm larvae have the highest lipid content in dry weight. This information is essential when choosing which insect to use, especially in recipes that replace some of the ingredients with insects. Knowing the predominant macronutrient content allows the use of insects more strategically, according to their specific nutritional needs.

**Figure 1:**
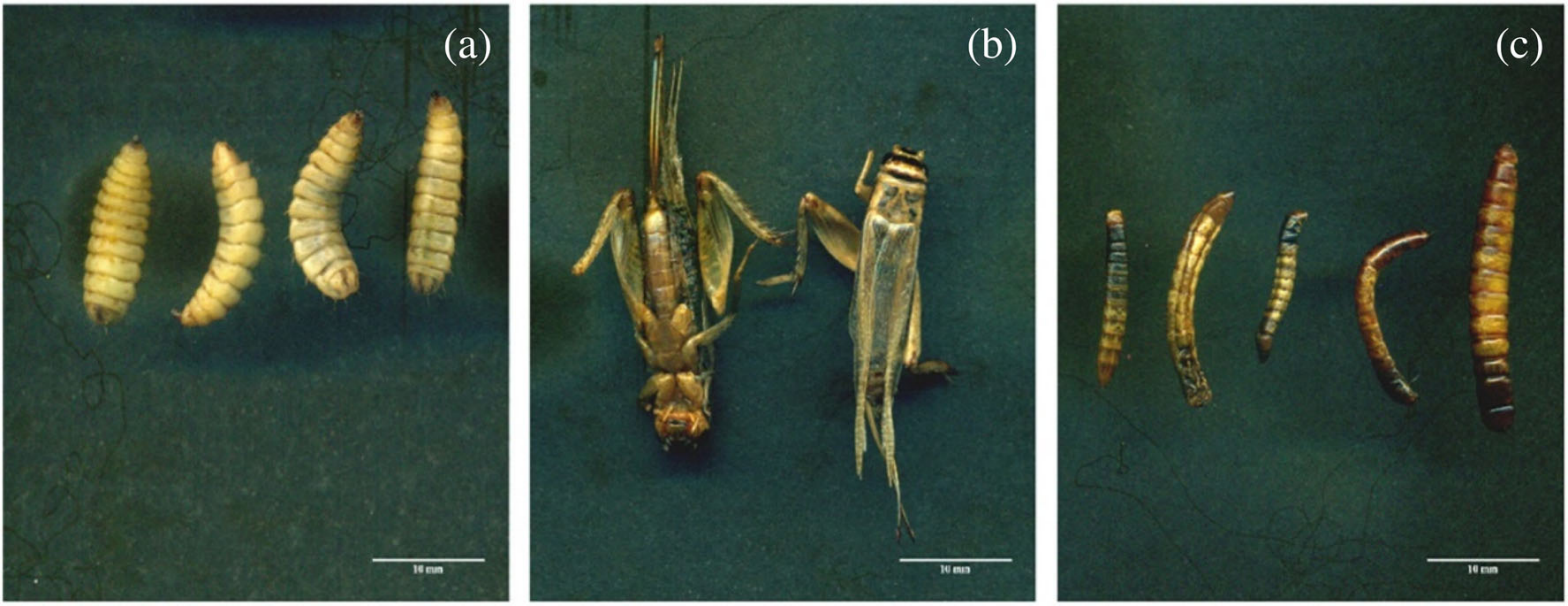
Insects. (a) *Hermetia illucens*; (b) *Acheta domesticus*; (c) *Tenebrio molitor*. Source: González *et al*.^35^

Figure 1 illustrates the differences in insect morphology, which influence, for example, the crude protein content and the proportion of essential amino acids. In the study conducted by González *et al*.,^35^ the species *H. illucens* and *Tenebrio molitor* were analyzed in their larval stage, whereas *Acheta domesticus* was considered in their adult stage. Compared with those of the other species, the *Acheta domesticus* flour presented the highest protein content.

### Environmental benefits

The breeding of insects for human consumption offers significant benefits for environmental preservation, such as lower water consumption, a reduction in greenhouse gas emissions, and a more efficient food conversion rate, in addition to contributing to a reduction in deforestation. This is because the amount of space required for insect production is much smaller than that required for monoculture and livestock practices, which are traditionally used in food production. For example, to produce 1 kg of mealworms (*Tenebrio molitor*), only 18 m^2^ are needed. Although the exact amount of water needed to produce different species of insects is not yet fully known, it is estimated to be much lower than that required for the production of traditional meats. To produce the same amount of beef, 254 m^2^ and 22 000 L of water are needed, which can reach 43 000 L; for pork, 63 m^2^ and 3500 L are needed; and for chickens, 51 m^2^ and 2300 L are needed.^36^, ^37^

A study by Huis *et al*.^38^ revealed that crickets need less than 2 kg of feed to gain 1 kg of body weight. In comparison, the amount of feed required to increase body weight by 1 kg is approximately 2.5 kg for chickens, 5 kg for pork and up to 10 kg for beef. Up to 80% of the mass of many edible insects is used, suggesting a significant reduction in food loss, unlike what occurs in cattle, pigs and chickens, where only 40% and 55% of the mass is ingested and digested by humans.^39^

The production and consumption of food of animal origin have significant environmental impacts, especially in the case of ruminants. When the environmental effects of different animal production systems, such as global warming potential, are compared, protein from insects has emerged as a promising alternative to reduce the environmental impact of human consumption.^40^ Insects can meet the growing demand for animal protein without requiring large areas of deforestation. In addition, if insects are consumed directly as food, they may play an important role in reducing greenhouse gas emissions throughout the food chain.^41^

Zielińska *et al*.^42^ reported that insects emit fewer greenhouse gases per unit of protein than traditional livestock; in addition, insect digestion results in lower methane emissions, an extremely potent greenhouse gas. The creation of insects generates lower levels of ammonia, a pollutant often associated with livestock, which is responsible for 14.5% of greenhouse gas emissions.^43^

Insect farming is an economic practice that promotes the use of food by‐products, creating a sustainable cycle in which the by‐products serve as food for the insects themselves. In this model, leftovers from the food industry, such as fruit peels, vegetable scraps and other organic waste, are reused, reducing waste and promoting efficiency in the use of resources. Insects, which feed on these by‐products, have become excellent sources of protein and nutrients for both human consumption and animal feed.^17^, ^44^

### Nutritional value

Insects are highly nutritious and are increasingly recognized as promising sources of proteins, lipids, minerals and vitamins. Their nutritional composition varies widely according to species, developmental stage (eggs, larvae, pupae or adults), diet, geographic origin and sex.^17^ In general, proteins and lipids are the predominant macronutrients, while insects also provide relevant amounts of micronutrients such as iron, zinc and vitamin B12.^45^ As summarized in Table 1, edible insects often appear nutritionally comparable to conventional protein sources; however, such comparisons must be interpreted with caution.

**Table 1:** Comparative nutritional composition of edible insects and conventional protein sources (per 100 g edible portion)^a^

Food source	Protein (g)	Fat (g)	Iron (mg)	Calcium (mg)
Mealworm larvae (*Tenebrio molitor*)	~24	~5	~2.2	~23
Chicken breast (cooked)	~32	~3	~0.5	~6
Beef (lean, cooked)	~26	~12	~2.7	~13
Fish (salmon, cooked)	~23	~4	~0.6	~45
Lentils (cooked)	~9	~0.4	~3.3	~19

^a^ Data compiled from US Department of Agriculture (USDA) FoodData Central and Rumpold and Schlüter^46^; values are indicative and depend on species, processing and moisture content.

Edible insects are frequently described as protein‐rich foods, with reported protein contents ranging from approximately 20 to 76 g per 100 g on a dry‐weight basis. Direct comparison with meats, fish and legumes may be misleading when differences in moisture content (fresh weight *versus* dry matter) are not considered, as insects generally contain less water than muscle foods, inflating apparent protein density on a fresh weight basis. Moreover, protein content is often overestimated due to the widespread use of the conventional nitrogen‐to‐protein conversion factor (N × 6.25), which does not adequately account for chitin‐derived non‐protein nitrogen. Recent evidence indicates that insect‐specific conversion factors closer to 5.3 provide more accurate estimates of true protein content. Although insects generally exhibit relatively high protein digestibility (75–98%), this remains slightly lower than that of animal‐derived proteins such as eggs, beef and casein, but higher than that of most plant proteins.^47^

Beyond total protein content, protein quality is a critical nutritional parameter. Several insect species present favorable essential amino acid profiles, being particularly rich in lysine, threonine and tryptophan, and are capable of meeting recommended dietary requirements for growth and maintenance.^47^, ^48^, ^49^ Nevertheless, robust data on protein quality remain limited, as most insect proteins have not been evaluated using standardized *in vivo* methods such as protein digestibility‐corrected amino acid score (PDCAAS) or digestible indispensable amino acid score (DIAAS). Instead, available evidence relies largely on *in vitro* digestibility assays, which limits direct comparison with conventional animal proteins. Consequently, nutritional equivalence between insects and traditional protein sources cannot yet be conclusively established.

Protein digestibility and amino acid bioavailability are further influenced by chitin, a dietary fiber forming the insect cuticle and exoskeleton that may account for up to 50% of total carbohydrates. Chitin can reduce protein utilization, although its partial or complete removal has been shown to improve digestibility. Additionally, chitin may trigger inflammatory or allergic responses in sensitive individuals by activating chitinase expression.^17^

The total lipid content of insects ranges from approximately 2% to 62%, depending on species, developmental stage and diet. In many species, lipids are characterized by a high proportion of unsaturated fatty acids, including omega‐6 and omega‐3 fatty acids such as oleic, linoleic and α‐linolenic acids, which contribute to membrane integrity, modulation of inflammatory responses, cardiovascular health and neurological function.^50^, ^51^ However, lipid composition is strongly influenced by rearing substrates and processing, introducing variability and raising concerns regarding nutritional consistency and potential contamination.

Regarding micronutrients, several insect species contain relatively high levels of minerals such as iron, zinc and calcium, as well as B vitamins including riboflavin, pantothenic acid and biotin, sometimes exceeding levels found in conventional animal products.^52^ Nonetheless, information on micronutrient bioavailability remains scarce, and the presence of chitin and other matrix components may influence mineral absorption. Therefore, the nutritional relevance of these micronutrients cannot be reliably assessed based solely on compositional data.

Overall, Table 1 illustrates that edible insects have the potential to contribute meaningfully to dietary protein and micronutrient intake. However, compositional similarity to conventional protein sources does not necessarily translate into nutritional equivalence. Harmonized analytical methods, standardized data expression and well‐designed human studies are essential to accurately assess protein quality, nutrient bioavailability and the role of edible insects as alternatives to meat, fish or legumes.

### Safety and acceptance of insects in human food

Edible insects have attracted increasing interest as alternative protein sources; however, their inclusion in the human diet raises relevant safety and acceptance concerns that must be rigorously addressed. Despite their nutritional potential, microbiological, chemical, and allergenic hazards (Table 2), together with the absence of fully harmonized regulatory frameworks, remain key challenges for their safe and widespread incorporation into food systems.

**Table 2:** Main food safety hazards associated with edible insects.

Hazard type	Examples
Microbiological	*Salmonella*, *Listeria monocytogenes*, *Bacillus cereus*
Chemical	Heavy metals, pesticides, mycotoxins
Allergenic	Tropomyosin, chitin‐related cross‐reactivity

Insects naturally harbor diverse microbial communities that may include bacteria, fungi, and heat‐resistant spores capable of surviving conventional processing. Microbiological hazards include pathogenic species such as *Salmonella* spp., *Listeria monocytogenes*, *Bacillus cereus* and *Staphylococcus aureus*, which may proliferate during rearing, harvesting or post‐processing when hygienic conditions are inadequate.^53^, ^54^ Spore‐forming bacteria are of particular concern, as they may survive mild thermal treatments, highlighting the need for validated processing protocols. Heat treatments such as pasteurization or high‐temperature short‐time (HTST) processing can effectively reduce microbial loads when time–temperature combinations are properly optimized to ensure safety without compromising the nutritional quality of proteins and lipids.^55^ Accordingly, the implementation of hazard analysis and critical control points (HACCP) systems throughout insect production and processing is strongly recommended by FAO and European Food Safety Authority (EFSA) to ensure microbiological safety and traceability.

Chemical hazards are primarily associated with the bioaccumulation of heavy metals, including cadmium, lead, arsenic and mercury, as well as pesticide residues and mycotoxins. The extent of contamination depends on insect species, developmental stage, substrate composition and environmental conditions, with considerable interspecies variability reported.^56^ Although insects reared under controlled feeding conditions generally present lower contaminant levels than wild‐harvested specimens, concentrations may still approach or exceed the maximum residue limits established by EFSA and Codex Alimentarius. Mycotoxin contamination, such as aflatoxins, ochratoxin A and zearalenone, may occur through fungal growth in rearing substrates, making substrate selection, monitoring and storage critical control points for risk mitigation.

In addition to exogenous contaminants, some insect species contain endogenous anti‐nutritional or toxic compounds, including alkaloids, saponins, tannins, oxalates, phytates and cyanogenic glycosides, which may impair nutrient absorption or exert cytotoxic effects at high concentrations.^57^ Appropriate processing strategies, such as thermal treatment, fermentation and enzymatic hydrolysis, have been shown to substantially reduce these compounds and improve nutritional bioavailability.

Allergenicity represents one of the most significant safety concerns related to edible insects. Proteins such as tropomyosin and arginine kinase, as well as chitin and chitosan from the exoskeleton, are known to mediate cross‐reactivity with crustacean, mollusk and house dust mite allergens, posing risks for sensitized individuals.^58^ The EFSA has acknowledged that allergic reactions may occur following ingestion or inhalation of insect proteins, even after heat processing. Consequently, clear allergen labeling, risk communication and allergen management strategies analogous to those applied to crustacean‐based foods are essential to protect vulnerable consumers.

Regulatory recognition of edible insects varies globally. In the European Union, selected species (e.g., *Tenebrio molitor*, *Locusta migratoria* and *Acheta domesticus*) have been authorized as novel foods under Regulation (EU) 2015/2283 following EFSA safety assessments. In the United States, the Food and Drug Administration (FDA) regulates insects intended for human consumption under good manufacturing practices. Internationally, FAO and World Health Organization (WHO) emphasize the need for standardized microbiological criteria, hygienic design and traceability within insect farming systems to ensure consumer safety.^59^, ^60^

Beyond safety, cultural and psychological barriers significantly influence consumer acceptance, particularly in Western societies where insects are often associated with dirt or disease. Evidence suggests that presenting insects in processed or ‘invisible’ forms, such as flours, protein powders or incorporated ingredients, can reduce neophobia and increase willingness to consume insect‐based foods.^61^, ^62^ Acceptance is further shaped by perceived health benefits, environmental sustainability and openness to novel foods.^63^, ^64^ Strategic communication highlighting nutritional value, safety assurance and environmental impact reduction is therefore critical for building consumer trust.

Regulatory frameworks for edible insects differ substantially among jurisdictions. In the European Union (EU), insects are classified as novel foods under Regulation (EU) 2015/2283, requiring pre‐market authorization based on safety assessment by EFSA. In the United States, insects are regulated as conventional foods under FDA oversight, without a specific novel food category. In many other regions, regulatory guidance remains fragmented or absent, representing a barrier to large‐scale commercialization.

In summary, ensuring the safety of edible insects for human consumption requires an integrated approach encompassing validated microbiological controls (e.g., HACCP and optimized thermal processing), chemical and mycotoxin monitoring, allergen management and compliance with international regulatory frameworks. Simultaneously, improving consumer awareness and acceptance through education, transparent labeling and product innovation remains essential for the successful integration of insect‐based foods into mainstream markets.

### Insects as food ingredients

Given their diverse nutritional profiles, insects stand out as attractive food sources, especially given the increasing demand for sustainable and nutritious alternatives worldwide. Owing to their high protein content, they can be incorporated into recipes, partially or completely replacing conventional ingredients in the formulation of various foods.^4^

According to Meyer *et al*.,^56^ the nutritional composition and technofunctional properties of insect‐based ingredients may vary due to several factors, such as insect feeding during rearing; processing to obtain marketable flours; and additional treatments to extract valuable compounds such as fat, protein or chitin. The optimization of the diet and processing of insects are valuable strategies that should be investigated to improve the nutritional profile and functionality of insect‐based meals, in addition to facilitating their integration into recipes already known by Western consumers.^65^

The use of insects in food, especially bakery products, has been widely studied with the objective of enriching these products and improving their acceptance by consumers. Processed foods such as meatballs, sausages and hamburgers can also be prepared using insect protein. In some studies, researchers ground insects to obtain flour; in others, lipids were removed with organic solvents to create defatted flours, or insect proteins were extracted to produce protein concentrates or isolates, depending on the desired final protein concentration.^66^ Table 3 provides a detailed overview of the use of insects as food ingredients in a variety of products. This table highlights not only the different types of insects used but also their applications and improvements in food.

**Table 3:** Application of insect proteins as food ingredients

Insect	Food product	Improvements/benefits	Reference
*Schistocerca gregaria* and *Apis mellifera*	Protein concentrates	Superior foam stability compared to whey proteins.	^67^
*Ascra cordifera* and *Brachygastra melifica*	Protein concentrates	Concentration of essential amino acids within the recommendation established by FAO/WHO. Physical stability of emulsions.	^67^
*Tenebrio molitor*	Protein concentrates	Firm and fluffy foam with high stability and improved emulsification.	^68^
*Hermetia illucens* and *Apis mellifera*	Fermented dairy products	High fat and protein content.	^69^
*Tenebrio molitor* and *Bombyx mori*	Sausage	The cooking yield and firmness of the sausages increased.	^70^
*Zophobas morio*	Cooked sausages	Lower cooking losses and adequate emulsion stability.	^71^
*Tenebrio molitor*	Bread	Higher protein and fiber content.	^35^
*Gryllus assimilis*	Gluten‐free bread	More protein, higher porosity, higher lipid content and better performance in improving cohesion and elasticity	^72^

### Protein concentrates

**Figure 2:**
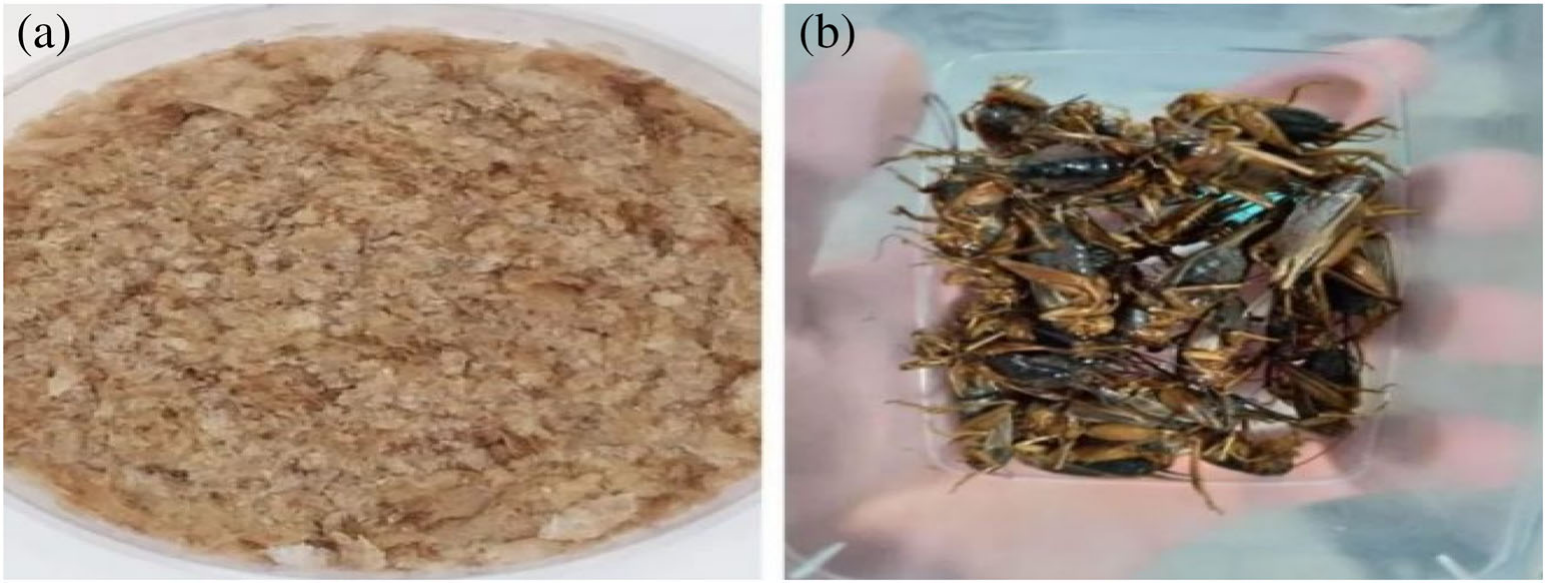
(a) Cricket‐based protein concentrate; (b) crickets used to make the protein concentrate. Source: Júnior *et al*.^74^

Protein concentrates are a viable alternative derived from various sources, such as dairy products, plants and insects. They are produced by removing non‐protein components from foods via alcoholic (aqueous) solutions such as 1‐butanol, isopropyl alcohol, and ethanol, in addition to acidic or basic solutions. Among the components removed are carbohydrates, minerals, antinutritional factors and low‐molecular‐weight nitrogen compounds.^73^ Figure 2 shows a protein concentrate made with crickets.

Mishyna *et al*. studied protein concentrates of *Schistocerca gregaria* (edible locusts) and *Apis mellifera* (bees) and reported that both exhibited emulsifying capacities similar to those of whey proteins, with high emulsifying stability after 2 h. The protein concentration of *Schistocerca gregaria* also presented a foam capacity similar to that of whey, with superior foam stability. In addition, the proteins of these insects had a balanced composition of essential and non‐essential amino acids, meeting the values recommended by the FAO. This study highlighted the great potential of insect proteins as substitutes for milk proteins in foods.^75^

Baigts‐Allende *et al*.,^67^ studied the extraction of insect protein concentrates from two Mexican species of edible insects, namely, *Ascra cordifera*, the adult juvenile jumil also known as bed bug, and *Brachygastra melifica*, the wasp larvae. They studied the protein structure and functionality (emulsifying capacity) and compared it with that of black soldier flies. The three concentrates obtained from the insects were considered to be of good nutritional quality because of their concentration of essential amino acids, which was within the recommendation established by the FAO/WHO for children. The emulsions stabilized by jumil concentrate and wasp larvae concentrate presented greater physical stability than black soldier fly larvae.

A study by Berthelot *et al*.,^68^ aimed to compare the approximate composition, protein profile, structure and foaming properties of two different mealworm protein concentrates (*Tenebrio molitor*): one with a residual lipid fraction and the other without detectable residual lipids. The researchers concluded that complete removal of residual lipids from a mealworm protein concentrate led to a modified particle size distribution and decreased surface hydrophobicity. It also significantly improved the protein foaming properties, leading to a firm and porous foam with high stability, in addition to showing improved emulsification.

Claims regarding the protein quality of insect‐derived ingredients must be supported by established quantitative indices and a clear description of the methods used to determine digestibility. The most widely used protein‐quality metrics are PDCAAS and DIAAS; complementary indicators such as NPU (net protein utilization) and BV (biological value) are also informative when available. PDCAAS is based on overall true/fecal protein digestibility and an amino acid score of 1.0, whereas DIAAS relies on the ileal digestibility of individual indispensable amino acids and is recommended by the FAO as more physiologically relevant for assessing protein quality. Because PDCAAS and DIAAS use different principles (fecal *versus* ileal; aggregate *versus* amino‐acid‐specific digestibility), authors should state explicitly which index was calculated, the reference amino‐acid pattern adopted (e.g., adult or young child), and provide the underlying digestibility coefficients or ileal amino‐acid digestibility data when available.^7^

Reported digestibility values for edible insects vary widely across species, life stages, substrates and processing conditions, with published crude protein digestibility ranges ranging from approximately 70–98% depending on the method and material examined.^58^ Methodological differences largely explain this variability: static *in vitro* digestion assays (e.g., INFOGEST‐like protocols) frequently yield higher apparent digestibility than ileal *in vivo* measurements unless *in vitro* results are calibrated against *in vivo* data.^47^ Therefore, whenever digestibility ranges or comparative claims (e.g., ‘comparable to casein/soy’) are reported, the manuscript must indicate whether values originate from *in vitro* or *in vivo* assays, whether ileal or fecal endpoints were used, the reference pattern for amino acid scoring, and whether the measurements refer to whole‐insect flours, defatted flours, protein concentrates, or hydrolyzed isolates.^10^, ^47^

Two analytical caveats require particular attention. First, chitin in the insect exoskeleton contains nitrogen, which is commonly included in crude protein calculations when the general conversion factor (N × 6.25) is applied; this practice can substantially overestimate the true protein content and, consequently, PDCAAS/DIAAS values. The use of species‐ or matrix‐specific nitrogen‐to‐protein conversion factors (or reporting protein as the sum of amino acids – ‘true protein’) is recommended to avoid artifactual inflation of protein quality indices.^47^ Second, the presence of antinutritional factors and protein–polysaccharide complexes (including chitin–protein associations) can reduce digestibility; thus, the retention or removal of chitin must be reported and considered when interpreting protein quality metrics.^10^

### Insect flour

**Figure 3:**
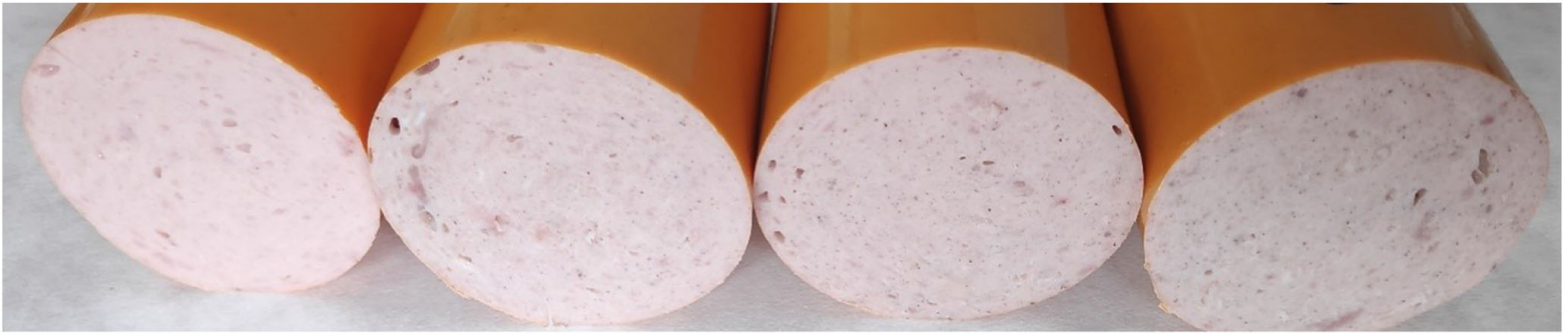
Cooked sausages with replacements of 5.0%, 7.5% or 10.0% lean pork – from left to right: control, 5.0%, 7.5% or 10.0% lean pork. Source: Vlahova‐Vangelova *et al*.^76^

Certain species of insects can be incorporated in powder form into products, offering additional nutrients that aim to improve the nutritional value of foods, correct nutrient or mineral deficiencies, and increase the foods beneficial health properties. Currently, many commercial food products are fortified with proteins from grains, such as rye or oats, and from legumes. In this context, insects stand out for being richer in protein than beans (23.5%), lentils (26.7%) or soybeans (41.1%), with levels ranging from 35% to 61% of this protein macronutrient.^70^ Figure 3 shows examples of sausages containing insect meal as an ingredient to replace lean pork.

Neves *et al*. investigated the use of black soldier fly (*H. illucens*) and bumblebee (*Apis mellifera*) flour as sustainable sources of protein in fermented dairy products, replacing milk powder. The addition of flours did not affect the nutritional, physical–chemical or microbiological composition of the product. The black soldier fly flour contained 23% fat and 40% protein, whereas the drone bee flour contained 26% fat and 33% protein. Both flours contain approximately 3% and 5% dietary fiber, respectively. The fat content of dairy products fermented with insect flour increased, but the protein (4.7%) and carbohydrate (13.4%) levels did not significantly change.^69^

Kim *et al*. evaluated the replacement of 10% lean pork meat in emulsion sausages with mealybug larvae (*Tenebrio molitor*) and pupae of silkworms (*Bombyx mori*).^70^ This substitution increased the cooking yield and firmness of the sausages, demonstrating the potential use of these insect meals as new protein ingredients. In a study by Scholliers *et al*., pork was partially replaced (5–50%) by meal of beetle (*Zophobas morio*) larvae in the production of cooked sausages. The use of 5% to 10% of this flour resulted in lower cooking losses and maintained adequate emulsion stability compared with sausages made exclusively with pork.^71^

**Figure 4:**
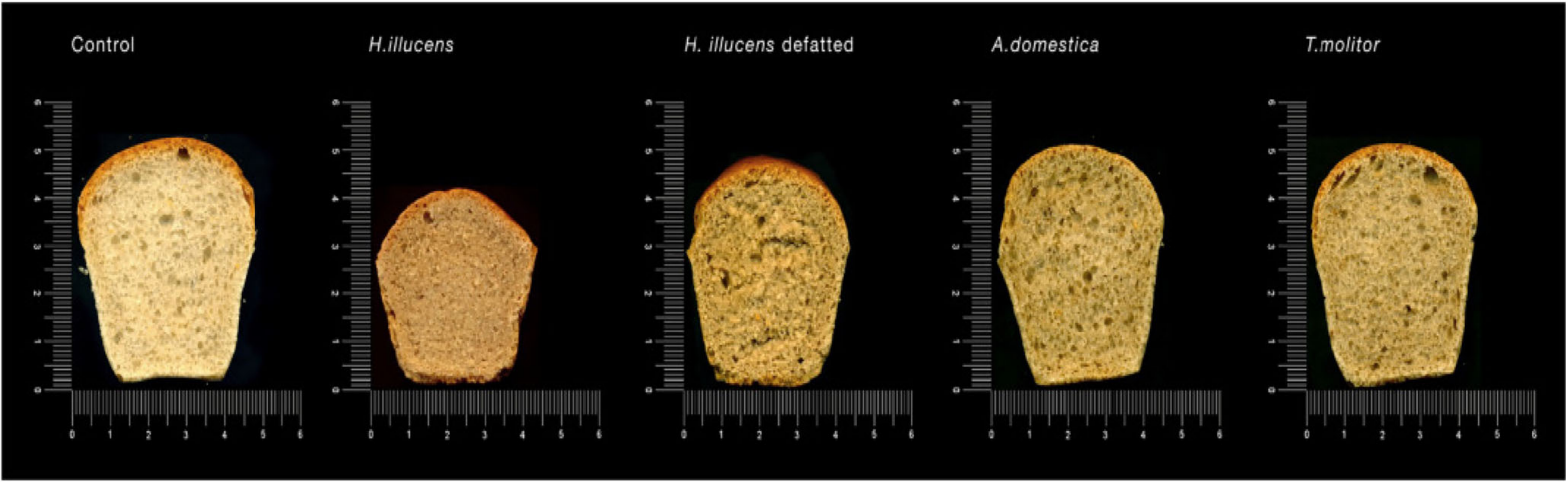
Cross‐sections of breads produced by replacing 5% wheat flour with different insect flours. Source: González *et al*.^35^

The inclusion of mealworm flour (*Tenebrio molitor*) in bread dough at replacement levels of 5 and 10% soft wheat flour (*Triticum aestivum*) was tested to produce breads fortified with protein. The bread fortified with 10% insect flour presented texture, color and specific volume parameters similar to those of the bread made with wheat flour, in addition to a higher protein and fiber content and a significant increase in the content of free amino acids. The breads enriched with 5% mealworm flour presented the highest specific volume and the lowest firmness.^35^ Figure 4 shows the breads made with 5% wheat flour substituted with different insect flours.

**Figure 5:**
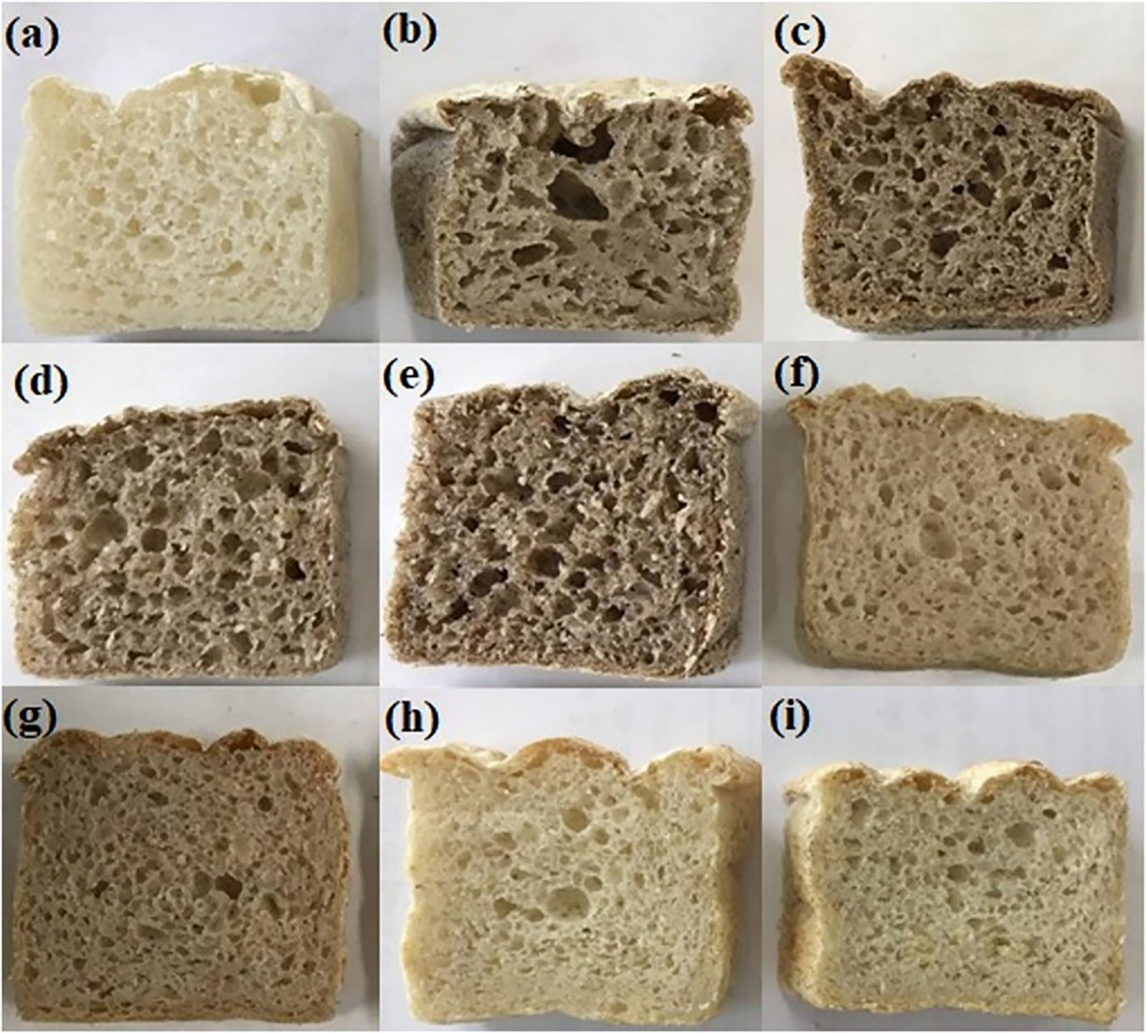
Images of the internal structure of bread slices: (a) control, (b) 10% cricket powder, (c) 20% cricket powder, (d) 10% oil‐free cricket powder, (e) 20% cricket powder without oil, (f) farinha de trigo sarraceno 10%, (g) 20% buckwheat flour, (h) 10% lentil flour, and (i) 20% lentil flour. Source: Machado and Thys.^72^

The study by Machado *et al*. aimed to characterize cricket powder (*Gryllus assimilis*) through physicochemical and microbiological analyses and evaluate its effects on the technological properties of gluten‐free breads (Fig. 5), comparing it with those of legume flours (lentils) and pseudocereals (buckwheat). Three protein sources were tested (cricket powder, lentils and buckwheat) in proportions of 10% and 20% based on rice flour and corn starch and were compared with a control sample without protein. The cricket powder presented 62.76% protein, 20.96% lipids, and 8.42% dietary fiber, among other components. Compared with the other flours, the cricket powder had greater water and oil retention capacities. The addition of cricket powder increased the hardness, chewiness and cohesion of the bread, resulting in a product with greater protein and lipid contents and better elasticity. The addition of 10% cricket powder increased the protein content by 40%, with more than 2.5 times more lipids, maintaining good quality without significant changes in crust color. These results indicate that cricket powder can be used to enrich gluten‐free breads, maintaining their sensory and nutritional properties.^72^

Duda *et al*. investigated the addition of cricket powder to pasta formulations to improve their nutritional value, texture and general characteristics. The macaroni without cricket powder served as a reference, while the formulations with 5%, 10% and 15% cricket powder were named CP5, CP10 and CP15, respectively. The addition of cricket powder significantly increased the protein content, from 9.96% in the reference sample to 16.92% in CP15 sample. There was also an increase in the levels of fat (from 1.31% to 4.73%) and minerals (from 0.86% to 1.46%). The samples enriched with cricket powder presented a darker color, similar to that of the whole pasta, and better firmness. The cooking weight decreased, and the mass loss during cooking decreased, but the cooking time increased.^77^

Akande *et al*. aimed to evaluate the impact of replacing skim milk with 15% locust bean pupae and silkworm powder in high‐energy cookies as alternative protein sources. Compared with locust beans, silkworm pupae presented higher levels of protein, fat and energy, which, in turn, stood out for being richer in fiber, ash and carbohydrates. The biscuits enriched with insects did not present obvious technical defects, such as problems with width, thickness or weight. Nutritionally, these cookies had slightly less moisture, protein and crude fiber than did the cookies made with skim milk but presented higher ash concentrations. In addition, the microbiological qualities were within safety standards, making the cookies safe for consumption. The study concluded that locust bean and silkworm pupal powders are suitable protein sources for high‐energy cookies.^78^

**Figure 6:**
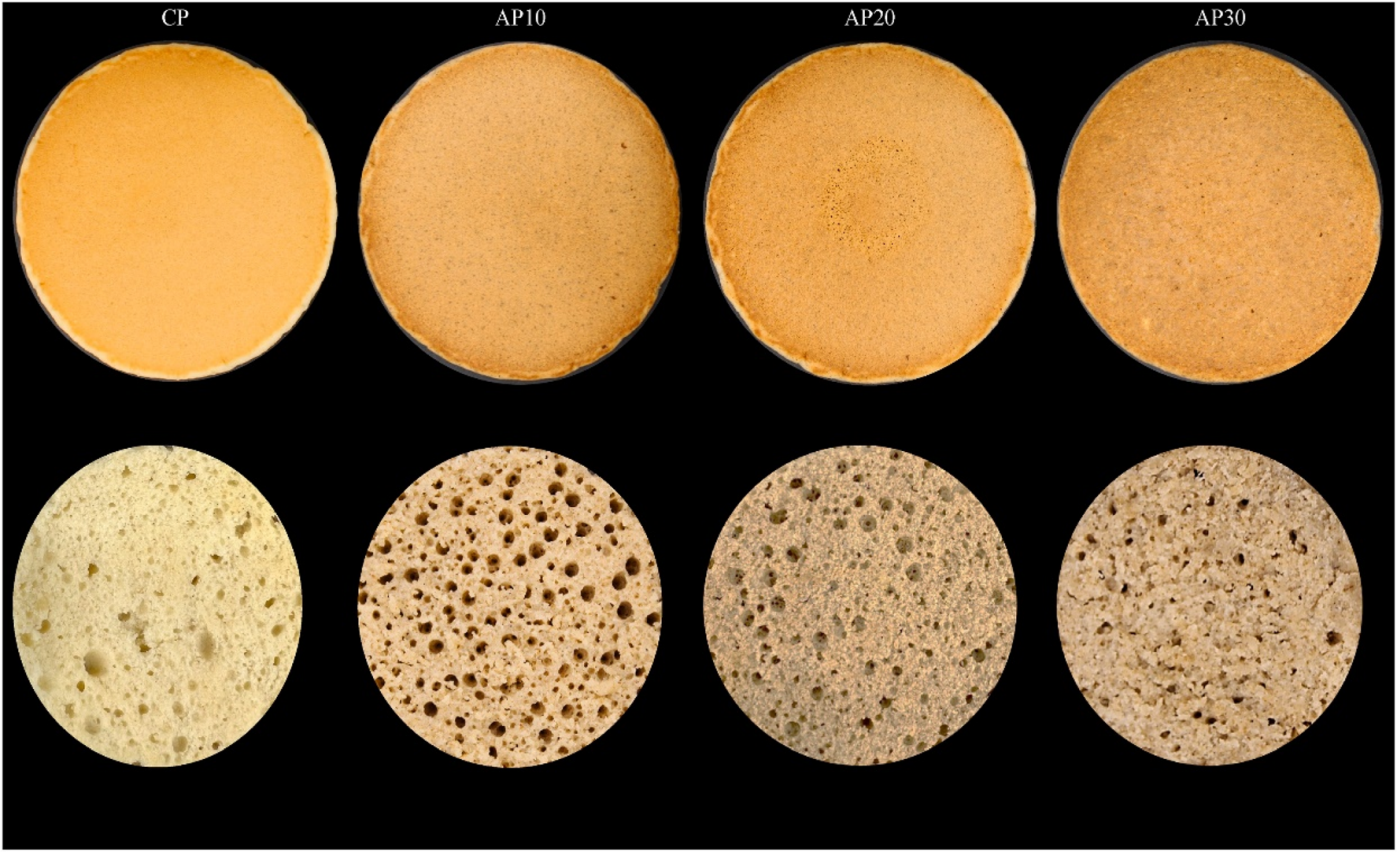
Cross‐section and top of the control sample (CP) and pancakes with increasing levels of domestic cricket flour (*Acheta domesticus*) called AP10, AP20 and AP30. Source: Bruttomesso *et al*.^79^

Bruttomesso *et al*. evaluated the rheological, technological and compositional properties of pancakes enriched with *Acheta domesticus* (AP) at proportions of 10%, 20% and 30%, respectively (Fig. 6). The pancakes were prepared with wheat flour and dry ingredients (sugars, salt and yeast), mixed with water, and the egg whites were beaten separately. The AP10, AP20 and AP30 formulas replaced the wheat flour with 10%, 20% and 30% AP, respectively. Compared with the control samples, the pancakes with different substitution ratios presented significant differences in hardness, chewiness and cohesiveness. The addition of insect powder increased the levels of fiber, protein and fat, with the AP30 formula resulting in a 52.9% increase in fiber. There were also changes in pore density and circularity, with reduced pore circularity in the enriched formulations.^79^

The objective of the study by Ronoh *et al*. was to evaluate the impact of fortifying wheat and wheat‐sorghum flours with *Ruspolia differens* powder (RDP) in cookies via substitutions of 5%, 15%, 25% and 40% RDP. The results showed that cookies made with 100% sorghum flour had a rough and dry texture, which could be unattractive to consumers. By combining sorghum flour with wheat flour, it was possible to improve the texture. In addition, the gelatinization temperature of the flours increased, whereas the viscosity decreased with increasing RDP. The cookies darkened as the RDP proportion increased, and there was a reduction in weight, lightness and characteristics such as elasticity and cohesiveness of the dough. The preference of consumers was greater for wheat crackers with 5% RDP than for wheat‐sorghum crackers with 0% RDP, and crackers made with 100% wheat were also well accepted.^80^

A study by Saetae investigated the impact of the Eri silkworm pupal protein extract on the quality of chocolate ice cream at concentrations ranging from 1% to 5% of the extract. The ice cream formulation included other ingredients, such as a stabilizer, sucrose, dextrose, cocoa powder, chocolate flavor and whole milk. Protein was the main component of the pupae, representing approximately 55.8% of the dry weight. The addition of the protein extract reduced the degree of overrun (volume of incorporated air), increased the melting rate and viscosity and increased the color of the ice cream. However, the sensory evaluation did not reveal negative impacts on the appearance, flavor or taste of the product.^81^

Kim *et al*. prepared five brine solutions for injection into pork loins via different ingredients, such as ground whole larvae and supernatants. The solutions used were as follows: control (no insects), WB (insect the whole body), SS (buffered saline and supernatant), SR (buffered saline and residue), DS (distilled water and supernatant) and DR (distilled water and residue). The objective was to increase the meat weight by 40% with 5% sodium chloride (NaCl) brine. Several parameters, such as pH, color, protein solubility, water holding capacity and lipid oxidation, were analyzed. The DR treatment had the lowest pH, whereas the DS treatment had the highest pH. The insect treatments resulted in a lower shear force (indicating greater tenderness), greater water retention, lower lipid oxidation and a lower thiobarbituric acid reactive substances (TBARS) value (indicating lower oxidation). These effects suggest that insect extracts can improve the nutritional quality and shelf‐life of the product. The increase in tenderness was attributed to the softening effect of the digestive enzymes present in the insects.^82^, ^83^

### Application in healthcare

Entomotherapy, defined as the use of insects or insect‐derived products for therapeutic purposes, has a long history in traditional medicine across diverse cultures. Insects and their preparations (fresh, cooked, dried, infusions, topical plasters and venoms) have traditionally been used to treat inflammatory conditions, infections and other ailments.^15^, ^36^, ^83^ However, despite growing scientific interest, the current body of evidence supporting therapeutic claims remains heterogeneous and largely preliminary. To avoid overstatement, claims regarding therapeutic efficacy should be explicitly framed according to the level of evidence, progressing from *in vitro* studies to animal models, small‐scale human studies and, ultimately, controlled clinical trials.

Most experimental research to date has focused on preclinical investigations. Numerous *in vitro* studies have demonstrated that insect extracts and isolated compounds, including peptides, enzymes, polyphenols, chitin derivatives and venoms, may exhibit antimicrobial, antifungal, antiviral, antioxidant, immunomodulatory and cytotoxic activities against cancer cell lines.^84^, ^85^ *In vivo* studies using animal models have reported anti‐inflammatory effects, such as reduced edema or decreased inflammatory biomarkers following administration of insect‐derived extracts or venoms, as well as other physiological responses.^86^, ^87^ Although these findings provide mechanistic insights, they do not constitute evidence of clinical efficacy and cannot be directly extrapolated to humans, particularly given limitations related to non‐physiological exposure levels, insufficient pharmacokinetic and toxicological characterization and interspecies differences in metabolism.

Human data on entomotherapy are scarce, with the notable exception of maggot debridement therapy, which has well‐established clinical applications in wound management. Outside this context, available human evidence is largely restricted to small pilot studies or short‐term dietary interventions. For instance, a randomized crossover study involving a limited sample size (*n* = 20) reported alterations in gut microbiota composition and reductions in circulating TNF‐α following cricket powder consumption.^88^ In addition, some traditional formulations, such as decoctions derived from *Coridius chinensis*, have been evaluated in small clinical series; however, these studies frequently lack adequate controls, standardized preparations and sufficient statistical power.^87^, ^89^ While such observations are hypothesis‐generating, suggesting, for example, potential prebiotic effects of chitin‐containing fibers or modulation of inflammatory markers, they do not demonstrate clinically meaningful outcomes such as disease prevention, symptom remission or improved survival.

Accordingly, statements such as ‘has anticancer properties’ or ‘is effective in treating X’ should be reformulated to accurately reflect the level of evidence. More appropriate expressions include ‘shows cytotoxic activity against cancer cell lines in vitro’ or ‘reduced tumor growth in preclinical models,’ accompanied by explicit clarification that no controlled clinical trials have demonstrated therapeutic efficacy in humans to date.

The therapeutic use of insects or insect‐derived products also requires careful safety evaluation. Key concerns include allergenicity due to cross‐reactivity with crustacean and house dust mite allergens, potential toxicity arising from environmental contaminants or endogenous insect toxins, and microbiological hazards associated with non‐sterile preparations.^52^, ^54^ Despite reported traditional uses of *C. chinensis* in Chinese medicine for conditions such as pain, gastrointestinal discomfort and disorders associated with yang deficiency, these applications should be interpreted within a cultural and historical context rather than as evidence of clinical effectiveness.

In summary, although entomotherapy represents a promising source of bioactive compounds and has deep ethnomedical roots, the current scientific evidence base, largely confined to *in vitro* and animal studies, does not support broad clinical recommendations. Rigorous, well‐designed human clinical trials, together with standardized preparations and comprehensive safety assessments, are essential before therapeutic claims can be substantiated.

## CONCLUSION

Edible insects represent a promising complementary protein source capable of contributing to the growing global demand for sustainable and nutritionally adequate foods. They provide high‐quality protein and relevant amounts of macronutrients and micronutrients, often comparable to or exceeding those of conventional animal‐derived foods, while offering clear environmental advantages. Nevertheless, their integration into human diets requires a cautious and evidence‐based approach. Current limitations include substantial variability related to insect species, developmental stage, rearing substrate and processing methods, as well as methodological heterogeneity in the assessment of protein content, digestibility and bioavailability. Inconsistencies in analytical approaches, particularly regarding digestibility protocols and the handling of chitin‐derived nitrogen, hamper comparability across studies and may bias protein‐quality indices such as PDCAAS and DIAAS. Moreover, most evidence supporting biological activities (e.g., antioxidant, anti‐inflammatory and cytotoxic effects) is derived from *in vitro* and animal models, while human data remain limited to small, short‐term studies and are insufficient to substantiate health or clinical claims. Food safety concerns, including allergenic cross‐reactivity, microbial contamination and chemical residues, together with regulatory heterogeneity and persistent barriers to consumer acceptance, further constrain large‐scale application. To support responsible uptake, regulatory authorities and industry stakeholders should prioritize the harmonization of guidelines for rearing substrates, hygienic production, analytical methods and labeling, including clear allergen disclosure. The implementation of robust quality‐management systems (good manufacturing practices (GMPs)/HACCPs), validated processing protocols and transparent communication regarding origin, processing and safety is essential to build consumer trust and enable the sustainable incorporation of insect‐based ingredients into food systems.

## AUTHOR CONTRIBUTIONS

Pamela Barroso de Oliveira: conceptualization, methodology, software, data curation, writing – original draft. Hugo Calixto Fonseca: data curation, writing – review and editing. Cláudia Regina Vieira: writing – review and editing. Gleidson Giordano Pinto de Carvalho: writing, review and editing. Jane Sélia dos Reis Coimbra: writing, review and editing. Bruna Mara Aparecida de Carvalho Mesquita: writing – review and editing, supervision, funding acquisition, project administration.

## CONFLICT OF INTEREST

The authors declare that they have no known competing financial interests or personal relationships that could have appeared to influence the work reported in this article.

## Data Availability

Data sharing not applicable to this article as no datasets were generated or analysed during the current study.
